# Medium‐Term Study Results of Ultrasound‐Guided Radiofrequency Ablation for Thyroid Follicular Neoplasm With Low SUV in PET/CT

**DOI:** 10.1155/ije/5566118

**Published:** 2026-01-28

**Authors:** An-Ni Lin, Wei-Che Lin, Yen-Hsiang Chang, Chen-Kai Chou, Pi-Ling Chiang, Yueh-Sheng Chen, Cheng-Kang Wang, Sheng-Dean Luo

**Affiliations:** ^1^ Department of Diagnostic Radiology, Kaohsiung Chang Gung Memorial Hospital, Chang Gung University College of Medicine, Kaohsiung, Taiwan, cgu.edu.tw; ^2^ Department of Radiology, Jen Ai Chang Gung Health, Taichung, Taiwan; ^3^ Department of Nuclear Medicine, Kaohsiung Chang Gung Memorial Hospital, Chang Gung University College of Medicine, Kaohsiung, Taiwan, cgu.edu.tw; ^4^ Division of Endocrinology and Metabolism, Department of Internal Medicine, Kaohsiung Chang Gung Memorial Hospital, Chang Gung University College of Medicine, Kaohsiung, Taiwan, cgu.edu.tw; ^5^ Department of Otolaryngology, Kaohsiung Chang Gung Memorial Hospital, Chang Gung University College of Medicine, Kaohsiung, Taiwan, cgu.edu.tw

**Keywords:** 18F FDG PET/CT, follicular neoplasm, radiofrequency ablation, thyroid gland, ultrasound

## Abstract

**Background:**

This study aimed to evaluate the medium‐term outcomes of radiofrequency ablation (RFA) for thyroid nodules with cytology of follicular neoplasm and low standard uptake value (SUV) in a positron emission tomography (PET/CT) study.

**Methods:**

Between January 2018 and January 2021, 40 patients diagnosed with follicular neoplasm underwent ultrasound, fine needle aspiration (FNA), or core needle biopsy (CNB) before RFA. PET/CT scans were performed in 34 patients before treatment. RFA, conducted under local anesthesia with an 18‐gauge internally cooled electrode and RF generator, was followed by evaluations of nodule volume modifications via ultrasonography, changes in symptomatic and cosmetic scores, and assessment of complications during and after the procedure. Six to twelve months post‐RFA, 33 patients received FNA for reassessment.

**Results:**

Significant volume reductions were observed during follow‐up, comparing values before RFA and at 6 months post‐RFA (7.31 ± 12.83 cm^3^, *p* < 0.001). The mean volume reduction ratios at 6 months and final follow‐up were 71.5% and 81.45%, respectively. The mean follow‐up time was 2.38 ± 0.9 years. Complications included vocal cord palsy and ptosis in one patient each, both recovering after RFA. No post‐RFA hypothyroidism was reported. Positive correlation was found between pre‐RFA thyroglobulin levels and PET/CT SUVmax values (*p* = 0.001).

**Conclusions:**

RFA is a safe and effective treatment for patients with low‐risk follicular neoplasm (SUVmax value < 5) in medium‐term follow‐up. For patients who are either ineligible for or prefer to avoid surgery, RFA presents a feasible alternative treatment option.

## 1. Introduction

Follicular cell–derived thyroid nodules share overlapping cytomorphological features, which are classified as follicular neoplasm (FN) or suspicion of FN (SFN), based on the Bethesda category system [1, 2]. The diagnosis of FN incorporates various pathologies, including follicular adenoma, adenomatoid nodules of multinodular goiter, follicular carcinoma, follicular variants of papillary carcinoma, Hürthle cell adenoma, and Hürthle cell carcinoma. Between approximately 15% and 30% of nodules within this category prove to be malignant. Nevertheless, the fine needle aspiration (FNA) cytology of an FN does not differentiate between benign and malignant tumors [3]. Ultrasonography (US) is the initial imaging technique applied to evaluate the morphologic features of thyroid nodules. In previous studies, ultrasonographic characteristics, such as solid, hypoechoic, microcalcifications, and intranodular vascularization were considered to be predictors of malignancy, although with inconclusive results [4, 5]. In addition, molecular testing tools such as mutational testing, including BRAF, RAS, RET/PTC, and PAX8/PPARγ, have been suggested to assess malignancy risk and facilitate the clinical decision‐making process [6]. However, the application may be limited due to cost and further restricted by access to suitable laboratory facilities. Hence, the core needle biopsy (CNB) has been recognized as a more effective diagnostic instrument for FN compared to FNA. However, when it comes to guiding the necessity for a diagnostic thyroidectomy, CNB does not show any significant advantage over repeated FNA [7]. Diagnostic thyroidectomy is often recommended to achieve a definitive diagnosis.

Positron emission tomography/computed tomography using glucose analog fluorine‐18 fluorodeoxyglucose (18F‐FDG PET/CT) is characterized by a higher glucose metabolic rate in thyroid malignancies than in normal thyroid tissues or benign tumors [8–11]. In thyroid nodules with intermediate FNA cytology, 18F‐FDG PET/CT has been applied to differentiate benign from malignant thyroid nodules and to avoid unnecessary diagnostic thyroid lobectomies [12]. The overall cutoff value for benign thyroid lesions reported in previous reports ranges from 2 to 5 [13–17]. Therefore, lesions with SUVmax below 5 are generally considered low‐risk.

Radiofrequency ablation (RFA) is a nonsurgical, minimally invasive technique, which has been widely used to treat benign thyroid nodules. Patients with recurrent thyroid cancer for curative or palliative purposes, and patients with primary thyroid cancer who refuse or are ineligible for surgery, may choose RFA as an alternative treatment [18]. According to current guidelines, surgery remains the standard treatment for FN on FNA or CNB [6]. However, there is currently limited literature available concerning the application of RFA in FNs. One study of 10 patients with FNs of < 2 cm having received treatment by RFA showed no recurrence in a 5‐year follow‐up period [19]. A separate study involving 22 patients who had low standard uptake value (SUV) FN underwent RFA, with none of the patients presenting with local tumor recurrence, lymph node metastasis, or distant metastasis during the follow‐up period [20]. Both studies indicated that RFA may be a safe and effective alternative management for patients who refuse surgical intervention.

The aim of this study was to evaluate the medium‐term outcome of RFA for the treatment of FN with low metabolic activity (SUVmax < 5) on 18F‐FDG PET/CT and to explore the correlation between metabolic parameters and laboratory findings. Although most patients exhibited low SUV values, a few borderline cases with slightly higher uptake were also analyzed to provide a more comprehensive assessment of RFA efficacy in real‐world clinical settings.

## 2. Materials and Methods

### 2.1. Patients

This retrospective study was approved by the Institutional Review Board of Chang Gung Medical Foundation (IRB No. 202201391B0), and the IRB approved the waiver of the participants′ consent. Data were retrospectively collected between January 2018 and January 2021. The patient inclusion criteria were as follows: [1] diagnosed FN or SFN based on FNA or CNB [2]; the presence of clinical symptoms or cosmetic issues [3]; thyroid nodule without suspicious malignant features such as spiculated margin, microcalcifications, and taller than wide or lymph node metastasis on US [4]; no evidence of distant metastasis in 18F‐FDG PET/CT or US [5]; and refusal of primary surgery or its contraindication. The study primarily focused on nodules with low metabolic activity (SUVmax < 5) on PET/CT, representing low‐risk lesions. A few borderline cases with SUVmax slightly above 5 were included because they met all other low‐risk clinical and cytological criteria, allowing a more complete evaluation of RFA outcomes. After a comprehensive explanation to patients of the potential risks of undetected malignancy and of the need for regular follow‐up to detect early tumor regrowth, RFA was performed in 40 patients.

### 2.2. Preablation Assessment

The pre‐RFA clinical assessment encompassed the evaluation of nodule‐related symptoms, cosmetic appearance, and thyroid function. Patients’ nodule‐related symptoms were evaluated using a specialized questionnaire that concentrated on five key clinical symptoms: compression, cough, difficulty in swallowing, voice changes, and pain. Each identified symptom contributed one point to the overall score, generating symptom scores that varied between 0 and 5. The cosmetic appearance score was quantified on a scale of 0–3, where a score of 0 denoted no noticeable or tactile mass, 1 signified a mass that was palpable but not visible, 2 pointed to a mass only visible during swallowing, and 3 corresponded to a readily visible mass.

Baseline laboratory data included measurements of serum thyroid–stimulating hormone (TSH), free thyroxine (fT4), triiodothyronine (T3), and thyroglobulin (TG) levels. These parameters were assessed to establish the initial status of the patients′ thyroid function.

The US, US‐guided FNA, and US‐guided CNB procedures were performed by an experienced radiologist, W.C.L., who has more than 15 years of expertise in the field. To obtain accurate measurements, three‐dimensional (3D) dimensions of each thyroid nodule were estimated based on their maximum lengths in the anterior–posterior, medial–lateral, and cranial–caudal directions. The tumor volume was then calculated using the following equation: *V* = π*abc*/6, where *V* represents the volume, and *a*, *b*, and *c* represent the largest diameter and the other two perpendicular diameters, respectively.

### 2.3. 18F‐FDG PET/CT

Patients were made to fast for 4 h before the procedure. They were then given a single bolus dose of F‐18 FDG (370‐440 MBq) via IV injection. After 1 h, head and neck F‐18 FDG PET/CT scans were taken using a PET/CT system (Discovery ST PET/CT system; GE Healthcare, Waukesha, WI, USA). Unenhanced CT scans were performed for correction and fusion with PET scans, which ran from head to upper chest (5 min per bed). PET images were corrected using CT scans and reconstructed to a resolution of 5.47 × 5.47 × 3.27 mm using the ordered subsets expectation maximization (OSEM) algorithm. Blood glucose levels were checked, and none exceeded 180 mg/dL. ROI was defined on the target lesion (thyroid nodule) in the transaxial PET images for semiquantitative analysis. The maximum SUV was calculated using the following criteria:

(1) SUV=tissue concentrationMBq/mLinjected doesmbq/patient′s body weightg.

Thyroid nodules possessing an SUVmax less than 5 were regarded as nonmalignant, whereas those with an SUVmax exceeding 5 were deemed potentially malignant [12, 20].

### 2.4. RFA

The RFA procedure was executed solely by one radiologist, W.C.L., and carried out as an outpatient service for every patient involved. A local anesthetic compound comprising of 2% lidocaine hydrochloride, sodium bicarbonate, and epinephrine was used at the incision point and around the thyroid gland. The selection of the electrode tip size was guided by the size of the tumor and its proximal vital structures. Using the trans‐isthmic approach, the electrode was carefully introduced into the thyroid nodule, prioritizing the farthest and deepest segment of the nodule. The RFA procedure was undertaken in real‐time, employing ultrasound guidance with the utilization of the moving shot technique. The conclusion of the ablation was ascertained when all theoretical ablation units of the nodule had transitioned into transient hyperechoic zones. Every patient was able to withstand the procedure, and after 60 min of meticulous observation alongside ice pack application and neck compression, they were discharged. As a standard protocol, patients were then directed to the otolaryngology department for vocal cord paralysis checks using flexible fiberoptic laryngoscopy.

### 2.5. Follow‐Up

Patients were scheduled for ultrasound and clinical assessments at the follow‐up visits occurring at the first, third, and sixth months, and every six to twelve months thereafter. In order to assess the status of the neoplasm, postablation FNA was conducted between six and twelve months post‐RFA. Serum tests were conducted during the follow‐up visits at the sixth and twelfth months. Alterations in the largest diameter of the nodule and the nodule’s volume were assessed via ultrasound. The rate of volume reduction was calculated using ultrasound imaging and the equation: volume reduction ratio (%) = (initial volume [mL] − final volume [mL]) ∗ 100/initial volume. Any major and minor complications were evaluated in accordance with the standard terminology set by the Society of Interventional Radiology (SIR) and were documented after the RFA procedure.

### 2.6. Statistical Analyses

The statistical analyses were performed using IBM SPSS, Version 21 (Armonk, NY). Continuous variables were shown as mean ± SD; categorical variables were calculated as frequencies or percentages. Alterations in the maximum nodule diameter, nodule volume, percentage volume reductions, and adjustments in symptom and cosmetic scores and serum tests throughout the follow‐up phase were analyzed using the Wilcoxon signed rank test and the mixed linear model. The correlation between nodular volume, SUVmax value, and TG level was determined utilizing the Spearman rank test. A *p* value of less than 0.05 was considered to represent statistical significance.

## 3. Results

### 3.1. Clinical Data

The clinical data of patients at enrollment and follow‐up are summarized in Table 1. The maximal tumor diameter and tumor volume prior to RFA were 3.01 ± 1.63 cm (range: 0.8–8.7 cm) and 10.05 ± 16.64 (range: 0.15–36.54 cm^3^), respectively. The mean nodule volume decreased significantly from baseline (10.05 ± 16.64 cm^3^) to 5.47 ± 12.89 cm^3^ at 1 month, 3.78 ± 7.32 cm^3^ at 3 months, and 2.84 ± 4.72 cm^3^ at 6 months after RFA (*p* < 0.001). The mean volume reduction ratios at the 1‐, 3‐ and 6‐month follow‐ups were 36.3%, 59.3%, and 71.5%, respectively. The mean follow‐up time was 2.38 ± 0.9 years (range: 0.76–3.81 years). The mean symptom and cosmetic scores were significantly reduced from baseline to the 6‐month follow‐up: from 0.39 ± 0.84 to 0.02 ± 0.15 (*p* = 0.043), and from 1.68 ± 1.29 to 0.64 ± 0.94 (*p* = 0.001), respectively. Serum fT4, T3, and TSH levels remained within normal limits prior to RFA and at the 6‐month follow‐up. However, there was a significant decrease in the TG level prior to RFA and 6 months after RFA (96.32 ± 160.92 vs. 13.48 ± 16.63; *p* < 0.001).

**Table 1:** Demographic data of patients with follicular neoplasm who underwent ablation.

Characteristics	Radiofrequency ablation (*n* = 40)	*p* value
Gender (male: female)	6:34	
Age (year)	45.1 ± 12.7	
Pretreatment pathology (follicular neoplasm: Hürthle cell neoplasm)	35:5	
PET SUVmax	2.49 ± 1.22	
TSH pre/post	1.28 ± 0.76/1.64 ± 1.12	0.047^∗^
Free T4 pre/post	1.10 ± 0.22/1.07 ± 0.15	0.253
Thyroglobulin pre/post	96.32 ± 160.91/13.48 ± 16.63	< 0.005^∗^
Nodule volume (cm^3^) pre/post	10.05 ± 16.64/1.97 ± 4.31	< 0.005^∗^
Symptomatic scale pre/post (0–5)	0.39 ± 0.84/0.02 ± 0.15	0.043^∗^
Cosmetic scale pre/post (1–4)	1.68 ± 1.29/0.64 ± 0.94	0.001^∗^
Follow‐up period (year)	2.38 ± 0.9	
Major complications	0/40	
Minor complications	2/40	
Recurrence	0	

^∗^ *p* < 0.05.

A total of 45 RFA sessions were performed in 40 patients. Post‐RFA FNAs were performed in 33 patients at 6–12 months after RFA to confirm a complete treatment, with a majority showing a Bethesda I or II category, except for one patient showing a Bethesda IV. Another session of RFA was arranged for this patient, with repeated FNA revealing a Bethesda category I, indicating no residual tumor. There were no major periprocedural complications, although a single patient experienced right vocal cord palsy and the other patient had ptosis, which both spontaneously recovered.

PET/CT was performed in 34 patients with FNs owing to its high negative predictive value in thyroid nodules of > 1 cm. There were three patients unsuitable for PET/CT due to small nodular size (< 1 cm), one patient due to advanced age, and one patient due to unwillingness. One patient was identified with a Bethesda category IV thyroid nodule at the initial survey, although this changed to a Bethesda category II with two subsequent aspirations. The mean SUVmax value of the PET/CT RFA patients was 2.47 ± 1.21 (range: 1.2–7.71). Although the study primarily focused on nodules with low SUVmax (< 5), a few borderline cases with slightly higher uptake were included because they met all other low‐risk imaging and cytological criteria. The pre‐RFA TG level was significantly positively correlated with an SUVmax value of PET/CT (*p* = 0.001), while the pre‐RFA TG level was not significantly correlated with the pre‐RFA nodule volume. No statistically significant correlations were found between the pre‐RFA nodule volume and the SUVmax value of PET/CT.

## 4. Discussion

### 4.1. Medium‐Term Results and Safety of RFA

**Figure 1:**
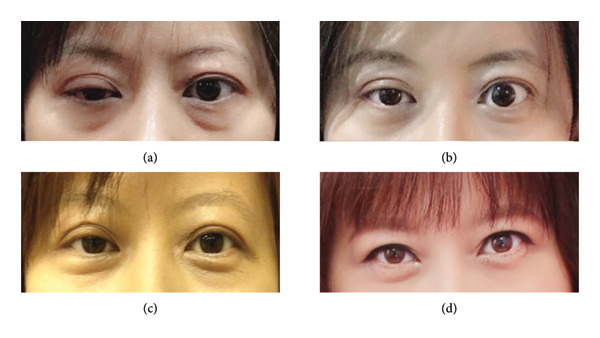
A 41‐year‐old female patient with a right follicular neoplasm presented for RFA. (a) Right eyelid ptosis was found approximately 12 h after RFA. Gradual recovery without additional treatment was observed at 7 days (b), 6 months (c), and 2 years (d) after RFA.

Our study demonstrates that RFA is not only effective in reducing nodular volume but also a safe treatment option for low‐risk FN (SUVmax value < 5) in medium‐term follow‐up. The complication rate was 5% (2/40; one patient with right vocal cord palsy who recovered within 3 months, and one patient with ptosis who recovered approximately 2 years after ablation) (Figure 1.), while no major complications were noted in our study. Postablated lesion was evaluated by FNA 6–12 months after RFA, and the majority of the cytology showed Bethesda category I or II. One patient had persistent Bethesda IV after the first RFA session, necessitating a second session. None of the patients presented with local tumor recurrence or lymph node metastasis.

### 4.2. The Relationship Between PET/CT and TG Level

TG is a glycoprotein which is synthesized by thyrocytes and released into the lumen of thyroid follicles with thyroid hormones [21]. Meanwhile, serum TG level represents a marker of persistent or recurrent disease in patients with total thyroidectomy or radioiodine ablation [22]. Although preoperative measurement of TG is not recommended for the initial evaluation of thyroid nodules, a serum TG level of 1000 μg/L seems to be a reasonable cutoff value in differentiating FN and follicular adenoma, with a sensitivity of 57% and a specificity of 87% [23]. In addition, in follicular or Hürthle cell neoplasm with a diameter of ≤ 2 cm, a preoperative serum TG level of > 80 ng/mL is an independent predictor of malignancy [24]. Furthermore, the concentration of TG not only indicates recurrence of malignancy but also implies the volume of differentiated thyroid tissue, inflammation status of the thyroid gland, and the magnitude of TSH stimulation [25]. Our results revealed a significant decrease in TG prior to RFA and 6 months after RFA, indicating that RFA is an effective treatment for FN.

Our study demonstrates a moderate correlation between the preablation serum TG level and SUVmax (Figure 2). However, there is no current literature focusing on the correlation of SUV in FN and serum TG level. The positive correlation between SUV of 18F‐FDG PET/CT and serum TG level in patients with histologically confirmed thyroid cancer has been evaluated in prior studies due to the increased disease burden [26, 27]. Of note, the sensitivity of 18F‐FDG PET/CT increases with an increased serum TG level. However, there is currently a lack of consensus with regard to the TG value at which the 18F‐FDG PET/CT accuracy is at maximum [27]. Further evaluation is thus required to determine the clinical impact and relationship between serum TG level and SUVmax in terms of FN.

Figure 2Correlation of maximal standardized uptake value (SUVmax) with thyroid nodule volume and thyroglobulin level. (a) No significant correlation was observed between thyroid nodule volume before RFA and thyroglobulin level. (b) Positive correlation of SUVmax with thyroid nodule volume (rs = 0.334, *n* = 33, *p* = 0.047). (c) Positive correlation of SUVmax with thyroglobulin level before RFA (rs = 0.576, *n* = 29, *p* = 0.001). (d) Positive correlation of SUVmax with thyroglobulin/TSH ratio before RFA (rs = 0.609, *n* = 29, *p* < 0.001, Spearman correlation coefficient).

**Figure:**
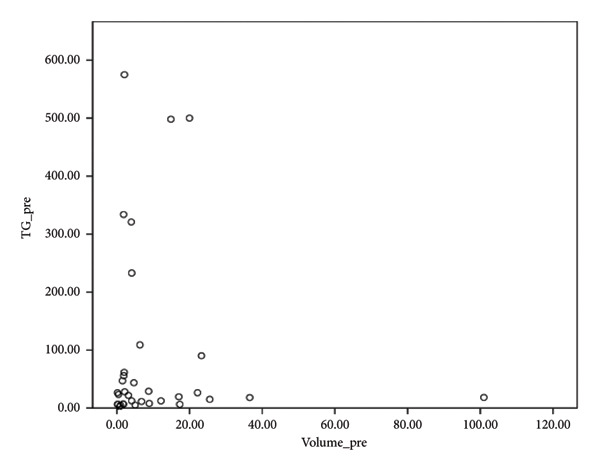
(a)

**Figure:**
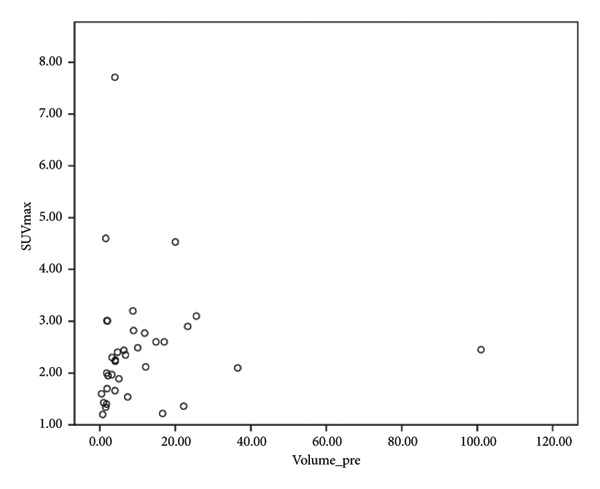
(b)

**Figure:**
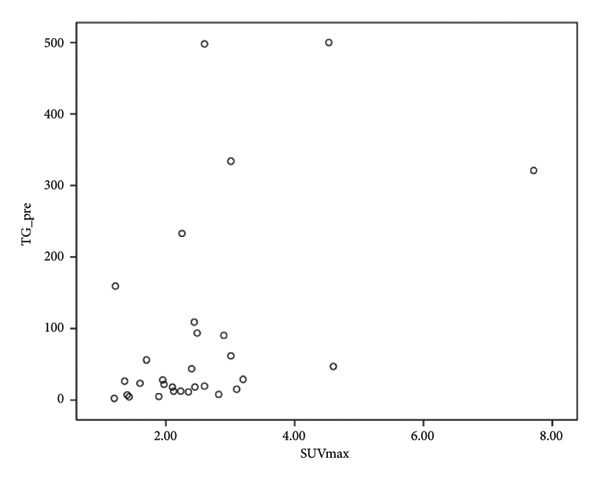
(c)

**Figure:**
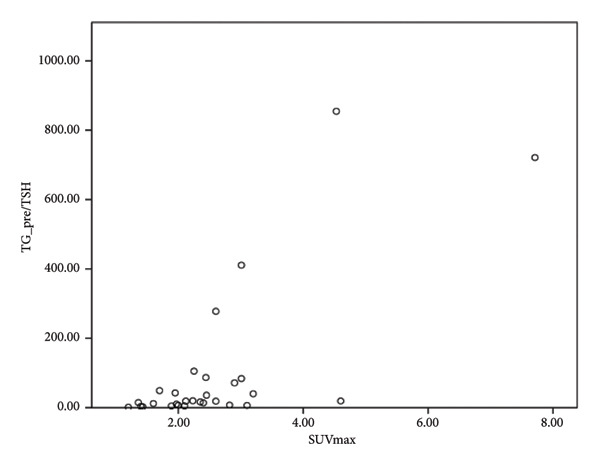
(d)

### 4.3. Follow‐Up in Patients Without Preablation PET/CT

A total of six patients in our study did not receive preoperative PET/CT for various reasons. Patient no. 18 refused examination due to personal circumstances, presenting with an initial thyroid nodular volume of 1.2 × 1.0 × 0.6 cm^3^ (0.36 mL) and a 2‐year follow‐up nodular volume of 0.5 × 0.4 × 0.5 cm^3^ (0.06 mL). While post‐RFA aspiration was not performed due to follow‐up being performed at another hospital, there was no evidence of local recurrence or lymph node metastasis during follow‐up. In addition, Patient no. 33 did not receive pre‐RFA 18F‐FDG PET/CT due to advanced age. Although thyroid cancer presents in all age groups, the mean age of thyroid cancer cases in Taiwan between 2013 and 2016 was 48.97 years, and it is a relatively self‐limiting disease in the elderly [28–30]. The volume reduction rate was 88.0% at the final follow‐up and repeated aspiration for the ablated lesion showed Bethesda category I. The other four patients presented with a nodular diameter of < 1 cm and were thus not suitable for PET/CT due to the relatively poor diagnostic accuracy of PET/CT in thyroid nodules of < 1 cm. None of these patients had nodular regrowth or lymph node metastasis during the follow‐up period.

### 4.4. RFA in Follicular Neoplasm With High SUVmax

**Figure 3:**
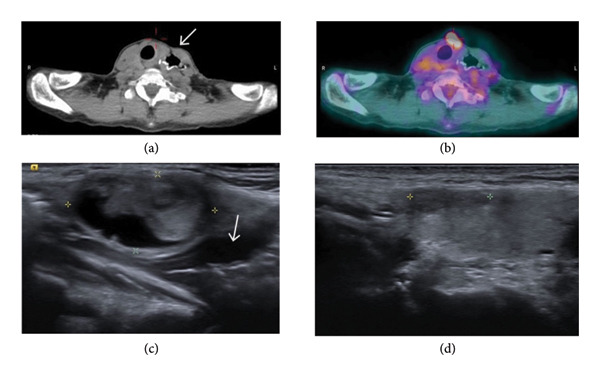
(a, b) PET/CT revealed a high SUVmax value of the left thyroid nodule with misregistration artifact and retained metallic clips at the reconstructed esophagus with colon interposition at the same anatomic level. Sonography of the same patient with follicular neoplasm before RFA (c) and 6 months after RFA (d) (arrow: reconstructed esophagus).

Our study indicates that RFA is not only an effective treatment for benign thyroid nodules but also a viable alternative treatment for FNs in patients not suitable or willing to undergo surgery. One 60‐year‐old female with a history of esophageal cancer came to our institution seeking a thyroid nodule evaluation. An echo‐guided CNB with results favoring Hürthle cell neoplasm was obtained. 18F‐FDG PET/CT revealed an SUVmax value of 7.71 in the left thyroid nodule with concurrent multiple hypermetabolic lung nodules and consolidative patches. The initial clinical impression was of thyroid malignancy with lung metastasis (Figure 3). However, a comprehensive tumor survey including serum tumor markers, image study, and CT‐guided biopsy for lung masses was performed with negative results. Although surgery is the first‐line treatment for suspicious thyroid malignancy, it was not recommended by the surgeon due to severe postoperative adhesion of the neck. After discussing the potential for malignancy with the patient, a single RFA session was performed without complication, with a significant nodular reduction rate (71.5%) observed during 6 months of follow‐up; furthermore, there was no evidence of residual tumor or tumor recurrence after 18 months of follow‐up.

### 4.5. Limitations

Several limitations to the present study exist. First, it is limited by the relatively small case number. Second, assessing the diagnostic accuracy of FN between FNA and CNB remains a matter of debate [31, 32]. Molecular testing, including BRAF, RAS, RET/PTC, and PAX8/PPARγ, has been reported to refine the probability of malignancy in FN or suspicious for FN cytology [6]. However, molecular testing may be unnecessary in low‐risk FNs, such as those presenting a tumor size < 30 mm, a tumor volume doubling rate < 1.0/year, and without suspicious features on sonography [33]. Last, due to the inherent limitations of retrospective single‐center studies, further prospective multicenter investigations with long‐term follow‐ups are needed to confirm the results.

## 5. Conclusion

RFA is a safe and effective treatment for patients with low‐risk FN (SUVmax value < 5) in medium‐term follow‐up. RFA should be regarded as a viable alternative treatment for patients not suitable or willing to undergo surgery.

## Ethics Statement

Ethics approval and consent to participate: This retrospective study was approved by the Institutional Review Board of Chang Gung Medical Foundation (IRB No. 202201391B0). Patient data were anonymized and treated with strict confidentiality to protect privacy. As a retrospective study, the IRB approves the waiver of the participants′ consent. We adhered to guidelines regarding the use of existing data while maintaining patient confidentiality. We will implement necessary measures to protect collected data and comply with data protection regulations. The study was conducted in accordance with the principles outlined in the Declaration of Helsinki and other relevant ethical guidelines.

## Consent

Please see the Ethics Statement.

## Conflicts of Interest

The authors declare no conflicts of interest.

## Author Contributions

An‐Ni Lin wrote the main manuscript. Wei‐Che Lin and Sheng‐Dean Luo were responsible for the conceptualization, review, and editing of the manuscript. Yueh‐Sheng Chen was responsible for developing the methodology. Chen‐Kai Chou, Pi‐Ling Chiang, Yueh‐Sheng Chen, and Cheng‐Kang Wang prepared the statistical analysis and figures. Wei‐Che Lin and Sheng‐Dean Luo contributed equally to this work.

## Funding

The authors received no financial support for the research, authorship, and/or publication of this article.

## Data Availability

The materials used in this study will be made available upon reasonable request. Researchers who require access to the materials may contact An‐Ni Lin at annie1126@cgmh.org.tw to discuss the requirements and procedures for obtaining access.
